# Divergent *Hd1*, *Ghd7*, and *DTH7* Alleles Control Heading Date and Yield Potential of *Japonica* Rice in Northeast China

**DOI:** 10.3389/fpls.2018.00035

**Published:** 2018-01-26

**Authors:** Jing Ye, Xiaojun Niu, Yaolong Yang, Shan Wang, Qun Xu, Xiaoping Yuan, Hanyong Yu, Yiping Wang, Shu Wang, Yue Feng, Xinghua Wei

**Affiliations:** ^1^State Key Laboratory of Rice Biology, China National Rice Research Institute, Hangzhou, China; ^2^College of Agronomy, Shenyang Agricultural University, Shenyang, China

**Keywords:** heading date, *japonica* rice, Northeast China, GWAS, genetic diversity, selection

## Abstract

The heading date is a vital factor in achieving a full rice yield. Cultivars with particular flowering behaviors have been artificially selected to survive in the long-day and low-temperature conditions of Northeast China. To dissect the genetic mechanism responsible for heading date in rice populations from Northeast China, association mapping was performed to identify major controlling loci. A genome-wide association study (GWAS) identified three genetic loci, *Hd1*, *Ghd7*, and *DTH7*, using general and mixed linear models. The three genes were sequenced to analyze natural variations and identify their functions. Loss-of-function alleles of these genes contributed to early rice heading dates in the northern regions of Northeast China, while functional alleles promoted late rice heading dates in the southern regions of Northeast China. Selecting environmentally appropriate allele combinations in new varieties is recommended during breeding. Introducing the early *indica* rice’s genetic background into Northeast *japonica* rice is a reasonable strategy for improving genetic diversity.

## Introduction

Rice (*Oryza sativa* L.) is a short-day (SD) plant in which flowering time (or heading date) is severely delayed under long-day (LD) conditions (Shimamoto and Yokoi, 2005). However, rice is widely cultivated in high-latitude regions, such as Italy, Japan, and Northeast China (Shrestha et al., 2014). Northeast China, with its fertile soil, vast plains and relatively low population density, contains 4.5 million hectares of farmland and, in 2014, produced 32.9 million tons of rice (16% of the total rice production of China). This region is the main rice production area and an important commercial food base in China (Wei et al., 2008). Since 2012, the area for rice cultivation has expanded in Huma (51°8′N), which is the most northern region for rice cultivation in the world and has 16 h days during the hottest month of the year. After domestication and with artificial selection, rice’s adaptability to higher latitudes has improved because of its reduced photoperiod sensitivity (PS) (Fujino et al., 2013; Yan et al., 2013).

Flowering time (or heading date), which determines the beginning of the reproductive cycle, is greatly affected by environmental conditions (e.g., day length and temperature) and is mainly determined by PS, temperature sensitivity and vegetative-growth duration (Shang et al., 2012). The photoperiod-related gene network has been widely studied and is the major genetic pathway controlling heading date in rice (Gao et al., 2014). Rice as a model plant for molecular genetic studies shares many homologous genes with Arabidopsis. For example, *HEADING DATE 3a* (*Hd3a*) and *RICE FLOWERING LOCUS T* (*RFT1*), which are homologs of *FLOWERING LOCUS T* (*FT*) in Arabidopsis, act as florigen genes to accelerate flowering (Tamaki et al., 2007; Komiya et al., 2008). *Hd3a* promotes heading under SD conditions, whereas *RFT1* is a major floral activator under LD conditions (Kojima et al., 2002; Komiya et al., 2009). The florigen-regulated network is mainly induced by *HEADING DATE 1* (*Hd1*) and *EARLY HEADING DATE 1* (*EHd1*) (Sun et al., 2014). *Hd1*, which is a homolog of *CONSTANS* in Arabidopsis, promotes flowering under SD conditions and represses flowering under LD conditions by regulating the expression of *Hd3a* (Putterill et al., 1995; Yano et al., 2000; Ishikawa et al., 2011). *EHd1*, encoding a B-type response regulator without any ortholog in the Arabidopsis genome, promotes flowering by inducing the expression of two florigen genes under both SD and LD conditions (Doi et al., 2004).

Several upstream regulatory factors controlling flowering under LD conditions have been identified. Among them, *GRAIN NUMBER, PLANT HEIGHT AND HEADING DATE 7* (*Ghd7*), which is an LD-specific repressor of *EHd1* expression, plays a crucial role in controlling yields, plant heights and heading dates in rice, simultaneously (Xue et al., 2008; Itoh et al., 2010). *DAYS TO HEADING 8* (*DTH8*), encoding an OsHAP3 subunit of a CCAAT-box binding protein (HAP complex), delays flowering by inhibiting expression of *EHd1* under LD conditions (Wei et al., 2010b; Yan et al., 2011; Dai et al., 2012). *Heading date 17* (*Hd17*), which is an ortholog of Arabidopsis *EARLY FLOWERING 3*, down-regulates *EHd1* expression by decreasing *Ghd7* expression and up-regulates *Hd1* expression by decreasing *OsGI* expression, resulting in accelerated flowering under LD conditions (Saito et al., 2012; Brambilla and Fornara, 2013; Yang et al., 2013). *Days to heading 7* (*DTH7*) encodes a pseudo-response regulator protein (OsPRR37) and inhibits *Hd3a* expression to suppress flowering under LD conditions (Koo et al., 2013; Li et al., 2015).

With the rapid development of technology and decreasing cost of sequencing, single nucleotide polymorphism (SNP) markers are now used widely to construct genetic maps, establish DNA finger printing and analyze linkage disequilibrium (Lindblad-Toh et al., 2000; Syvänen, 2001; Primmer et al., 2002). A genome-wide association study (GWAS) is a powerful tool to identify genes associated with the quantitative variation of complex traits (Burton et al., 2007; Huang et al., 2010). Recently, GWAS has been applied to crops, particularly rice, resulting in the identification of several new genes (Gu et al., 2015; Dong et al., 2016; Yano et al., 2016). However, identifying unknown genes in rice is still a challenge because of spurious and indirect associations, which are generated by the strong population structure and low rate of linkage disequilibrium decay (Gupta et al., 2005; Hamblin et al., 2011).

In this study, 244 representative *japonica* rice cultivars from Northeast China with an enormous level of phenotypic diversity in flowering time, were grown in three environments having a wide geographical span (from 18°N to 41°N) to investigate the mechanistic responses to photoperiod. We attempted to identify flowering gene(s) involved in controlling the rice heading date in Northeast China using GWAS. The association mapping between SNPs and heading dates showed that flowering times were largely determined by the natural variations in *Hd1*, *Ghd7*, and *DTH7*. Thus, *Hd1*, *Ghd7*, and *DTH7* were sequenced to estimate the distributions of different allelic combinations associated with rice adaptability in the different zones of high-latitude regions. The ability to predict heading dates and yield potentials could be used to produce hybrid progeny from parental rice varieties in this region and provide guidance for molecular breeding.

## Materials and Methods

### Plant Materials and Growing Conditions

A set of 244 *japonica* rice varieties cultivated in Northeast China (including Heilongjiang, Jilin and Liaoning Provinces) were obtained from the National Medium Rice Genebank at the China National Rice Research Institute (Supplementary Figure S1 and Supplementary Table S1). Rice plant were grown in three natural environments at Lingshui (18°32′N, 110°01′E) (winter of 2015 and 2016), Hangzhou (30°15′ N, 120°12′E) (2016) and Shenyang (41°48′N, 123°25′E) (2016) (Supplementary Figure S1A). A 6 × 6 block planting design was used for each cultivar, with three replicates.

### Phenotype

The panicle numbers per plant, spikelets per panicle and grain yields per plant were measured from three plants in the middle of a planting block. Days to heading (DTH) were recorded as the period from the sowing date to the time when the first panicle appeared from the node (Koo et al., 2013). The PS index was calculated as (DTH_shenyang_ - DTH_lingshui_)/DTH_Shenyang_ (Gao et al., 2014). Pearson’s correlation coefficient calculations and a two-way analysis of variance for heading dates in the four environments were performed using SAS version 9.4.

### Genotype

Total genomic DNA was extracted from the leaf samples of each accession, which had been grown in Lingshui in 2015. All the accessions were genotyped using Illumina custom designed arrays. Genotypes of these accessions were called using Genome Studio. After removing nucleotide variations with SNPs of low quality and minor allele frequencies < 0.05, we identified 21,198 SNPs, which were used further for GWAS analysis (Supplementary Table S2). We used MSU_version 6^1^ to identify SNP and gene positions.

### Population Genetic Analyses

To analyze the population structures of 244 varieties, the genetic components were calculated using Structure version 2.3 (Pritchard et al., 2000). In the analysis, the number of clusters (K) was set from 1 to 10 with a length of burn-in period of 10,000 steps and Markov chain Monte Carlo replications of 100,000 cycles, five runs independently for each *K*-value (Evanno et al., 2005). The optimal number of genetic clusters was identified when the Δ*K* was set at its maximum. Using the CLUMPP software, the results of all of the genetic components were combined to obtain the Q matrix, which was used for further association mapping (Jakobsson and Rosenberg, 2007). Major allele frequency, gene diversity, polymorphic information content and Nei’s genetic distance (Nei, 1972) were assessed using PowerMarker version 3.25 (Liu and Muse, 2005). The construction of a neighbor-joining tree was based on pairwise Nei’s genetic distances. The principal component analysis was estimated using the software NTSYSpc version 2.1 (Rohlf, 1988). The Kinship matrix was calculated based on SNP data using TASSEL version 4.0 (Bradbury et al., 2007). The heatmap of pairwise relative kinship values was constructed using the R package “Lattice.”

### Genome-Wide Association Study (GWAS)

For the GWAS, general linear (GLM) and mixed linear (MLM) models (with both Q and K matrices as covariates) were used. The observed *P*-values from marker-trait associations and the linkage disequilibrium parameter were calculated using TASSEL version 4.0 (Bradbury et al., 2007). The Manhattan plot and linkage disequilibrium block were constructed based on the results of TASSEL using Haploview 4.2 (Barrett et al., 2005). The Bonferroni-corrected threshold for the *P-*value was 1/21,198 (*P* = α/*n*, α = 1) and 0.05/21,198 (α = 0.05), with corresponding -log_10_(*P*) values of 4.33 and 5.63, respectively (Yang et al., 2014). The candidate genes at the identified loci were screened from the MSU Rice Genome Annotation Project Database^2^.

### Sequence Analysis

For PCR, primers for amplifying *Hd1*, *Ghd7*, and *DTH7* were designed based on the sequences of *japonica* cultivar Nipponbare from the Rice Genome Annotation Project Database^3^ using Primer3 (Rozen and Skaletsky, 2000). The primers for amplification and sequencing are listed in Supplementary Table S3. The 50-μl total PCR reaction volume contained 25 μl KOD FX PCR buffer, 10 μl dNTPs, 1.5 μl each 10 pmol/μl primer, 5 μl template DNA, 6 μl PCR grade water and 1 μl KOD FX. The PCR cycle conditions were as follows: initial incubation at 94°C for 2 min, 35 cycles of 98°C for 10 s, 55°C for 30 s and 68°C at 1 kb/1 min, followed by a final extension at 68°C for 7 min^4^. Then, PCR products were purified by TaKaRa MiniBEST Agarose Gel DNA Extraction Kit Ver.4.0^5^ and sequenced in both directions with an ABI 3730xl DNA Analyzer^6^. The sequences were assembled using DNAstar software. Sequences were aligned using the ClustalW method (Thompson et al., 2002), and a phylogenetic analysis using the neighbor-joining algorithm (Saitou and Nei, 1987) was constructed in MEGA version 5.05. Nucleotide diversity and two neutrality test parameters, Tajima’s D (Tajima, 1989), and Fu and Li’s D (Fu and Li, 1993), were calculated with DnaSP version 5.0 (Rozas et al., 2003).

## Results

### Genetic Characterization of *Japonica* Cultivars from Northeast China

We constructed a natural population composed of 244 *japonica* rice varieties that were selected from Northeast China. 50.8, 35.7, 10.7, and 2.9% of these varieties were selected from Liaoning, Heilongjiang, Jilin, and other provinces, respectively (Supplementary Figure S1C). The ancestry of these accessions can be traced back to four varieties, Liaojing5, Liaojing326, Songjing3, and Hejiang20 (Supplementary Table S1). After removing SNPs with minor allele frequencies less than 0.05 or greater than 20% missing data, only 21,198 SNPs were identified (Supplementary Table S2). These SNPs were distributed throughout all 12 chromosomes. The neighboring distances between 78.33% of the SNPs were less than 20 kb and only 3.6% of inter-SNP distances were greater than 80 kb (Supplementary Figure S2). The decay of the linkage disequilibrium with the physical distance between SNPs occurred at 550 kb (Supplementary Figure S3) when the cut off value of the linkage disequilibrium parameter was set to half its maximum (Huang et al., 2010).

**FIGURE 1:**
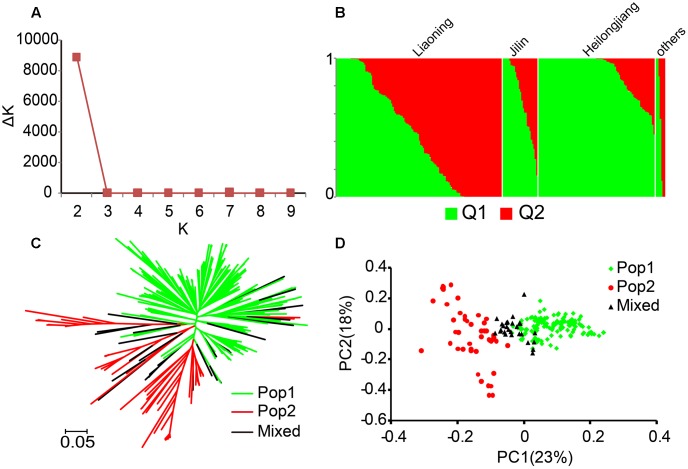
Population structure of 244 rice samples. **(A)** Δ*K* values plotted as the number of subpopulations. **(B)** Subpopulations inferred using Structure. **(C)** Neighbor-joining tree based on Nei’s genetic distances. **(D)** Principal component analysis. Values in parentheses indicate the percentage of variance in the data explained by each principal component.

Using these SNPs, we performed a genetic component analysis of each variety to quantify the population structure using Structure software. The Δ*K* achieved its maximum value when *k* = 2 (**Figure 1A**). Thus, the population was divided into two major subgroups and a mixed group based on the variety with *Q*-value >0.6 was appointed to corresponding Pop, and the rest were assigned to a mixed group. The geographic distribution analyses showed that all of the varieties of Heilongjiang and half varieties of Liaoning were assigned to Pop 1 (**Figure 1B** and Supplementary Table S4). Then, a neighbor-joining tree was constructed based on Nei’s genetic distance. The resulting tree showed two major divergent subgroups separated, with some overlapping (**Figure 1C**). The principal component analysis showed that these accessions were clearly separated into three sub-groups, indicating that the 244 cultivars were appropriately divided into two major sub-clusters (**Figure 1D**). Furthermore, the population differentiation statistics value between Pop 1 and Pop 2 was 0.1, indicating that a low level of subpopulation differentiation existed in the 244 *japonica* varieties (Supplementary Table S5). Finally, the inner relative kinship of Pop 1 was higher than that of Pop 2, and the relative kinships of the two groups both represented a strong relatedness (Supplementary Figure S4). Moreover, the genetic diversity (0.25) of Pop 1 was lower than that (0.29) of Pop 2, and both indicated a low genetic diversity (Supplementary Table S6). Thus, there were close genetic relationships in the present populations, especially in Pop 1.

### Phenotypic Variation in the Heading Date

All 244 *japonica* rice cultivars were grown in three distinct geographical locations, ranging from lower to higher latitudes, across China to investigate the mechanistic response to photoperiod (Supplementary Figure S1A). The correlation coefficients were both 0.80 under SD (2015 and 2016 in Lingshui) and LD (Hangzhou and Shenyang) conditions, while the remaining correlation coefficients were less than 0.7 (Supplementary Table S7). Two years of test data from Lingshui showed a similar pattern, thus we chose the 2015 test data without special mention for further analysis. A two-way analysis of variance revealed that the environment accounted for 78.15% of the variation in flowering time (Supplementary Table S8). Thus, environment had a dominant effect on flowering time.

**FIGURE 2:**
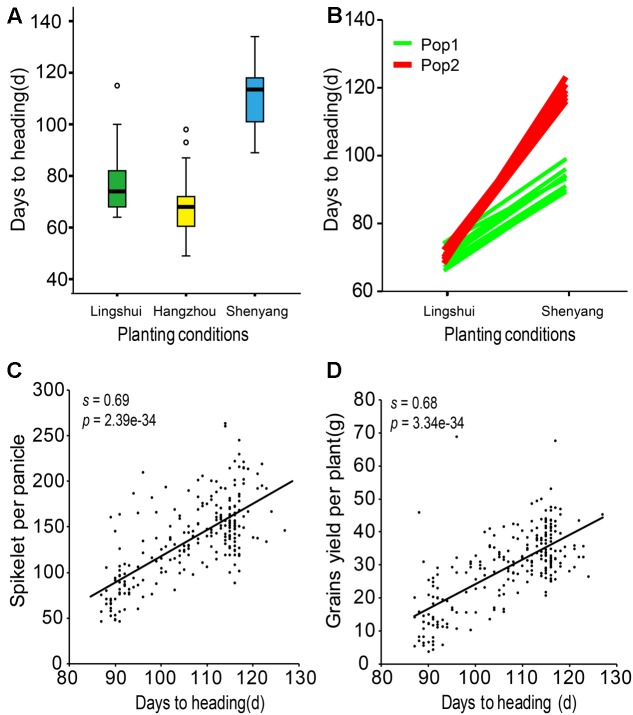
Association between grains and heading dates. **(A)** Heading dates of 244 accessions from Lingshui, Hangzhou, and Shenyang. Box edges represent the 0.25 and 0.75 quantiles with the median values shown by bold lines. Whiskers extend to data no more than 1.5 times the interquartile range, and the remaining data are indicated by asterisks. **(B)** Heading dates of a partial set of 244 accessions under natural SD (Lingshui) and LD (Shenyang) conditions. **(C)** Spikelets per panicle were associated with flowering time in Shenyang. **(D)** Grains per plant were associated with flowering time in Shenyang. The standardized coefficient is represented by s. Student’s *t*-tests were used to generate the *P*-values.

Compared with accessions in Hangzhou (LD and high temperatures), the heading dates were significantly later in Shenyang (LD and low temperatures), and accessions tended to flower later in Shenyang than in Lingshui (SD and mid-range temperatures) (**Figure 2A**). Thus, the varieties were highly sensitive to the environment. In addition, part of Pop 2 was more sensitive to day length than part of Pop 1 (**Figure 2B**). The molecular mechanisms of PS are addressed below. Finally, spikelet number per panicle and grain yield per plant correlated positively and extremely significantly with flowering time (**Figures 2C,D**). However, flowering time influenced yield mainly through the spikelet number per panicle (Supplementary Table S9).

### Association Mapping for Heading Date

**FIGURE 3:**
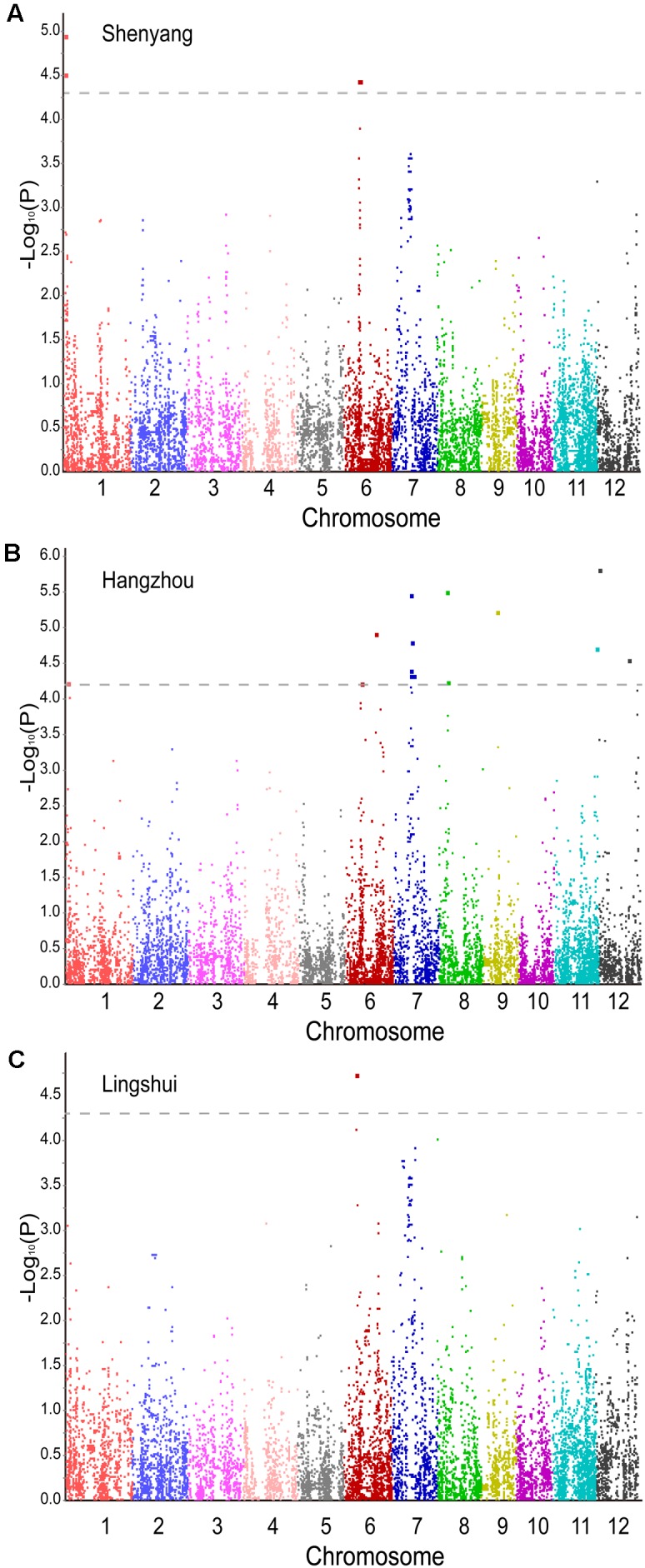
Manhattan plots of mixed linear models under three environmental conditions. **(A)** Shenyang. **(B)** Hangzhou. **(C)** Lingshui. The dashed line represents the significance threshold.

To choose a better model for trait-marker associations, GLM and MLM were used to perform the association analysis. The results of quantile–quantile plots of estimated -log_10_(*P*) showed that the MLM was significantly better than the GLM in controlling false positive (Supplementary Figure S5). Thus, for heading date, we used an MLM for the GWAS. Only a significant SNP (*P* < 2.36 × 10^-6^) was found for heading date in three environments (Supplementary Table S10). It may be too strict to use α = 0.05 for further analysis, so we examined the marginally significant association signals by using α = 1. A total of nine loci were identified as having *P* < 4.72 × 10^-5^ (α = 1) in a genome-wide scan of the three environments (two loci in Shenyang, six loci in Hangzhou, and one locus in Lingshui) (**Figure 3** and Supplementary Table S10). Among the nine loci, three were known associations and the other six were newly identified. The GWAS produced a similar result using phenotypic data collected in 2016 and 2015 in Lingshui (**Figure 3** and Supplementary Figure S6).

### Indirect Association Owing to Allelic Heterogeneity

**FIGURE 4:**
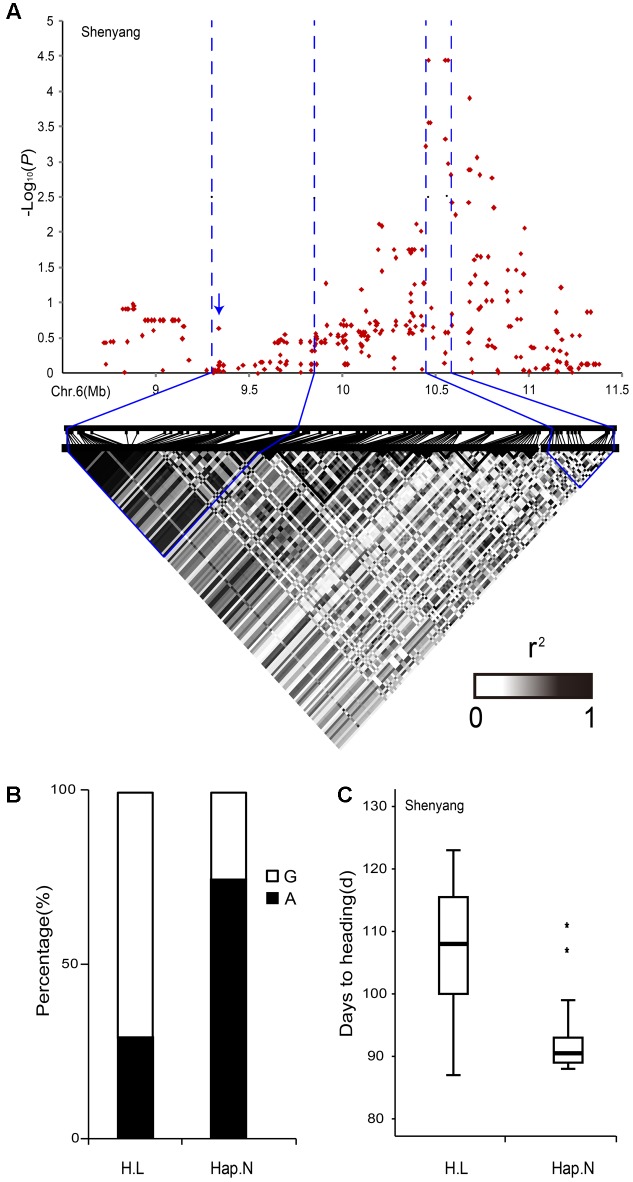
Analyses of the peaks for days to heading-associated haplotypes on chromosome 6. **(A)** Local Manhattan plot (top) and LD heatmap (bottom) surrounding the peak on chromosome 6. The arrows indicate the positions of nucleotide variations in *Hd1*. **(B)** The percentages of G base and A base corresponding to Hap.L and Hap.N, respectively, based on the SNP marker (R0610460215) in the highest peak. **(C)** Boxplots for days to heading based on Hap.L and Hap.N. ^∗^, outlier.

A peak on chromosome 6 was detected in both Shenyang and Lingshui, while the -log_10_(*P*) of the peak (4.31) in Hangzhou was very close to the marginally significant threshold (4.33). Using a linkage disequilibrium block analysis we obtained a 106-kb candidate region (10.46–10.57 Mb) (**Figure 4A**). However, no good candidate gene was found in this region, which was consistent with a previous study (Yano et al., 2016). By checking the region nearby, we observed that *Hd1* was localized in the next linkage disequilibrium block (9.22–9.61 Mb). There are various haplotypes of *Hd1*, including null, intermediate and fully functional alleles (Fujino et al., 2010), which may be the reason for the SNP markers could not achieve statistical significance in the region of *Hd1* (Yano et al., 2016).

All 244 accessions were sequenced for *Hd1* to confirm the mis-association and whether it corresponded to previous studies or not. In total, 14 haplotypes were identified for *Hd1* and some of them caused frame-shift mutations, which were likely to be non-functional (Supplementary Figure S7A). The average phenotypic values were clearly separated between the frame-shift mutations (Hap.F and Hap.L) and non-frame-shift mutations (Hap.B and Hap.N). In this analysis, we ignored other varieties containing minor haplotypes (Supplementary Figure S7B). The 14 haplotypes were divided into two clades, haplotypes of clade 2 mainly had large insertion and deletion fragments at position 512 of *Hd1* compared with clade 1 (Supplementary Figure S7C). Hap.L mainly corresponded to guanine, while Hap.N mainly corresponded to adenine in the highest peak (R0610460215) based on SNPs (**Figure 4B**). Thus, Hap.L and Hap.N were clearly different. The accessions carrying Hap.N showed earlier heading dates than accessions carrying Hap.L (**Figure 4C**). These results indicated that *Hd1* was a causal gene in this region and demonstrated that high polymorphism level of a gene may lead to indirect associations (Galichon et al., 2012).

### Indirect Association Owing to a High Linkage Disequilibrium Level

**FIGURE 5:**
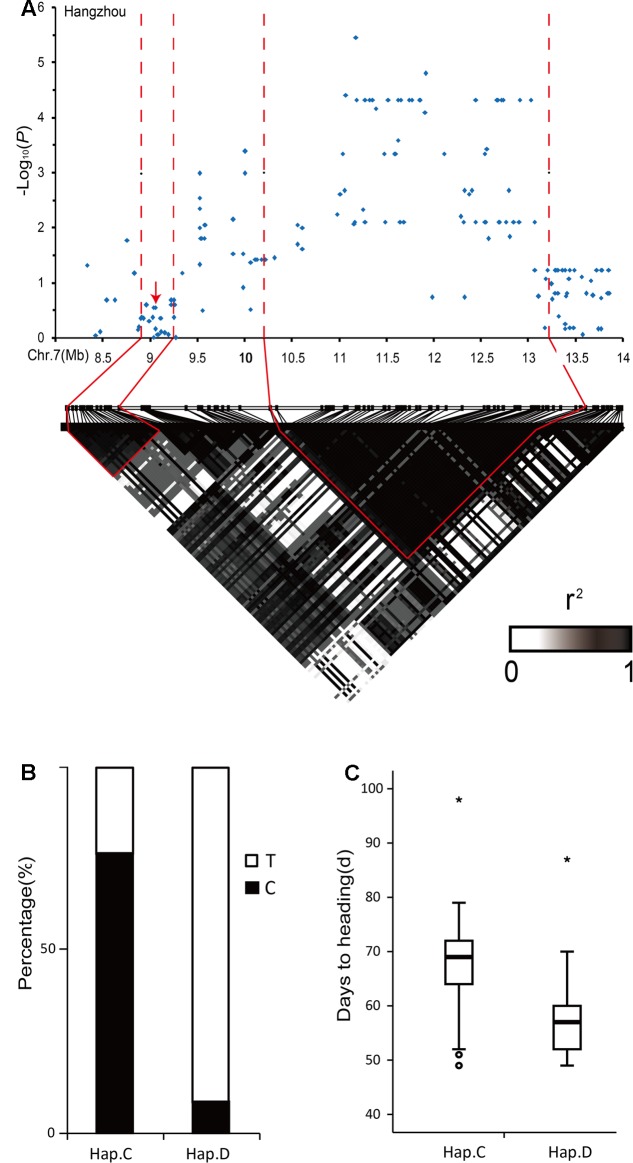
Analyses of the peak for days to heading-associated haplotypes in the centromeric region of chromosome 7. **(A)** Local Manhattan plot (top) and LD heatmap (bottom) surrounding the peak on chromosome 7. The arrows indicate the positions of nucleotide variations in *Ghd7*. **(B)** The percentages of T base and C base corresponding to Hap.C and Hap.D, respectively, based on the SNP marker (F0711913681) in the highest peak. **(C)** Boxplots for days to heading based on Hap.C and Hap.D. ^∗^, outlier.

The lead SNP in the centromeric region of chromosome 7 was specifically identified in Hangzhou, while its -log_10_(*P*) value exceeded 3 in Shenyang and Lingshui, and the value, as a compromised threshold, was used to detect the marginally significant association signals for GWAS (Feng et al., 2016). However, we could not find a good candidate gene in the region (10.22–13.21 Mb). When the candidate region covers a large distance (3 Mb) and is adjacent to the centromere, then the linkage relationships of the SNP markers are closer. Thus, we calculated the linkage disequilibrium block using a pairwise comparison of SNP markers separated by 5 Mb. This showed high level linkage relationship between the initial candidate region and an adjacent linkage disequilibrium block (8.91–9.25 Mb) (**Figure 5A**). In the linkage disequilibrium block, *Ghd7* was a key quantitative trait loci that simultaneously controlled yield, plant height and heading date in rice (Xue et al., 2008).

We identified four haplotypes containing Hap.D with a premature stop codon in the coding sequence of *Ghd7* (Supplementary Figure S8A). The varieties of Hap.D had earlier heading dates than the others because of premature stop codon (Supplementary Figure S8B). The two clades were separated from these four haplotypes, and Hap.C and Hap.D in the 244 varieties were concentrated in clade 2 (Supplementary Figure S8C). Hap.C and Hap.D mainly corresponded to cytosine and thymine, respectively, in the SNP marker (F0711913681) at the highest peak in this linkage disequilibrium block (**Figure 5B**). The accessions carrying Hap.C had later heading dates than accessions carrying Hap.D (**Figure 5C**). These results indicated that *Ghd7* was confirmed as a causal gene in this region and demonstrated that high levels of linkage disequilibrium may also cause indirect associations (Grady et al., 2011).

### Association Mapping Using GLM

**FIGURE 6:**
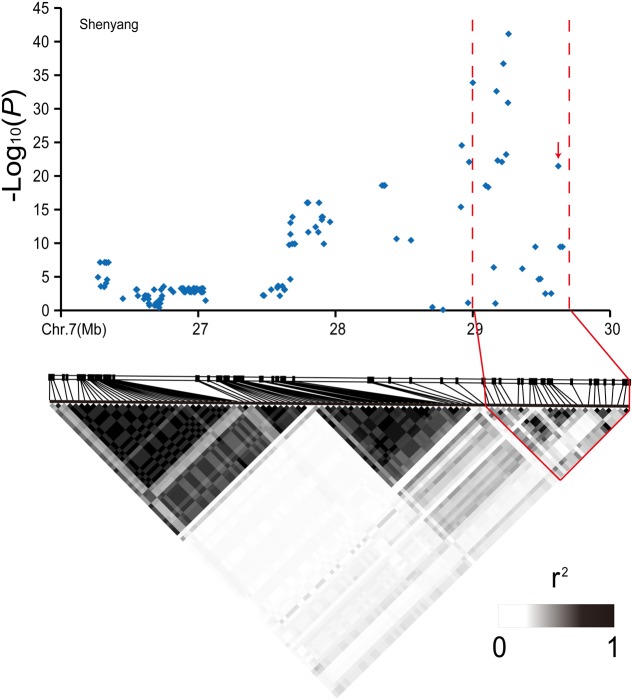
Analyses of the peak for days to heading-associated haplotypes at the very end of chromosome 7. Local Manhattan plot (top) and LD heatmap (bottom) surrounding the peak on chromosome 7. The arrows indicate the positions of nucleotide variations in *DTH7*.

Although the identification of heading date-related genes was effective using the MLM, some real genes may have been filtered out by the strict criteria. Therefore, association mapping was performed using the GLM. Two genes, *Hd1* and *Ghd7*, were identified using both MLM and GLM, while a locus on the distal end of chromosome 7 was only identified in Shenyang and Hangzhou using the GLM (**Figure 6** and Supplementary Figure S9). In the linkage disequilibrium block of the candidate loci, *DTH7* is strongly regulated by day length (photoperiod) and temperature (Gao et al., 2014). There were five haplotypes with distinct phenotypes. Varieties carrying Hap.C had a later heading date than plants carrying Hap.B or Hap.D (Supplementary Figures S10A,B). The haplotypes of the cultivars were divided into two clades (Supplementary Figure S10C). The vast majority of varieties carrying Hap.B or Hap.C and both of the haplotypes belonged to the same clades.

### Genetic Diversity among *Hd1*, *Ghd7*, and *DTH7*

To clarify whether *Hd1*, *Ghd7*, and *DTH7* had undergone selection during the expanded distribution and regional adaptation of *japonica* rice cultivars in Northeast China, we analyzed the nucleotide diversity (π) and the neutrality. The aligned lengths of exons that included insertion–deletions were as follows: 1,224 bp for *Hd1*, 774 bp for *Ghd7*, and 2,229 bp for *DTH7*. The π values for *Hd1* and *DTH7* were greater in Heilongjiang than in the other varieties, indicating more nucleotide diversity in Heilongjiang rice (Supplementary Table S11). However, *Ghd7* had similar π values in Heilongjiang (0.55) and Jilin (0.62). *Hd1* had the highest nucleotide diversity, especially in Heilongjiang (π = 5.52), while *DTH7* had the lowest variation in all regions. Tajima’s D and Fu and Li’s D statistics were not significant, except for those of *Hd1*, which were positive values.

### Function of Allelic Variations of *Hd1*, *Ghd7*, and *DTH7*

Alleles of *Hd1* and *DTH7* were classified into functional and non-functional types (Supplementary Table S12). Hap.F, G, L and N of *Hd1* are non-functional alleles owing to frame-shift mutations or insertions of large fragments (Takahashi et al., 2009; Zhang et al., 2015; Yano et al., 2016). In addition to the haplotypes reported by previous studies, three new alleles of *Hd1*, Hap.C, D, and E, showed loss-of-functions because of frame-shift mutations. The alleles of Hap.B and Hap.D were equal in effect to *PRR37-2* and *PRR37-1*, respectively, in *DTH7*, while the alleles of Hap.C and Hap.E were the same as *PRR37-2a* and *PRR37-1a*, respectively, having no functions (Koo et al., 2013). The alleles of *Ghd7* could be classified into strongly and weakly functional, and non-functional types. Hap.B and Hap.C of *Ghd7* corresponded to *Ghd7-1* and *Ghd7-2*, respectively, which were confirmed to be strongly and weakly functional, respectively (Xue et al., 2008). Hap.D of *Ghd7* was the same as *Ghd7-0a*, with no function.

### Regional Adaptation of the Three-Gene Combinations in Northeast China

The genetic effects of these three gene were clearly assigned to functional classifications. Thus, we investigated the effects of combinations of *Hd1*, *Ghd7*, and *DTH7* on heading dates in the present population (Li et al., 2015; Zhang et al., 2015). The combination of three non-functional loci (NNN) was associated with the earliest heading date under all conditions and was correlated with the lowest grain yield in Shenyang (**Table 1**). In general, the combinations functional *Hd1*, and non-functional *Ghd7* and *DTH7* (FNN), functional *Hd1*, weak functional *Ghd7*, and non-functional *DTH7* (FWN), and weak functional *Ghd7*, and non-functional *Hd1* and *DTH7* (NWN) exhibited similar heading dates, PS indices and grain yields under all three conditions. Functional *DTH7*, and non-functional *Hd1* and *Ghd7* (NNF) plants headed later and produced greater grain yields per plant than the plants with non-functional *DTH7* in Shenyang, where the day length is very long in the summer. However, there was little difference between heading dates of NNF plants and non-functional *DTH7* plants in Lingshui and Hangzhou. The heading dates were extremely delayed and grain yields were significantly increased in both non-functional *Hd1*, weak functional *Ghd7* and functional *DTH7* (NWF) and weak functional *Ghd7*, and functional *Hd1* and *DTH7* (FWF) plants. Moreover, plants with functional *DTH7* displayed significantly higher PS index values than plants with non-functional *DTH7*, indicating that functional *DTH7* was associated with a strong PS.

**Table 1:** Heading date and grain yield associated with japonica rice cultivars from Northeast China combinations of *Hd1, Ghd7*, and *DTH7*.

Combinations			Heading date(d)			PS index	Grain yield(g)
			
*Hd1*	*Ghd7*	*DTH7*	Lingshui	Hangzhou	Shenyang		Shenyang
N	N	N	68.10 ± 2.28	56.67 ± 4.65	92.71 ± 5.7	0.264 ± 0.04	18.25 ± 8.19
F	N	N	71.83 ± 12.84	57.67 ± 14.47	93.33 ± 9.31	0.235 ± 0.06	19.97 ± 8.82
F	W	N	70.33 ± 3.21	61.67 ± 1.15	94.67 ± 2.31	0.257 ± 0.03	19.51 ± 8.44
N	W	N	72.06 ± 8.85	59.30 ± 7.61	95.24 ± 7.45	0.244 ± 0.05	19.05 ± 7.14
N	N	F	69.67 ± 3.08	59.33 ± 3.93	99.5 ± 5.79	0.299 ± 0.03	29.29 ± 7.14
N	W	F	77.82 ± 7.63	69.07 ± 4.87	111.42 ± 7.12	0.300 ± 0.07	33.46 ± 9.37
F	W	F	77.00 ± 12.79	75.33 ± 11.99	114.44 ± 10.49	0.319 ± 0.15	34.27 ± 5.94


*N, non-functional haplotypes; F, functional haplotypes; W, weak functional haplotypes.*

**FIGURE 7:**
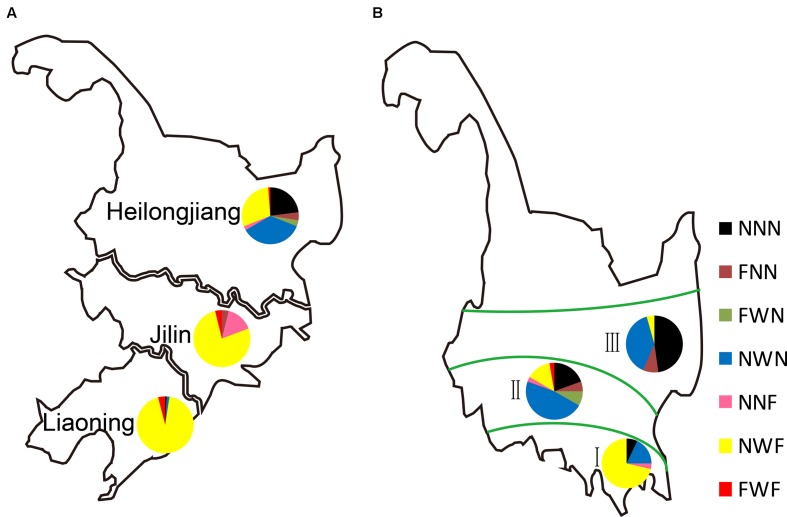
*Hd1*, *Ghd7*, and *DTH7* in rice haplotypes in Northeast China. **(A)** Regional adaptation of the three-gene combinations in Northeast China and **(B)** in three accumulated temperature zones of Heilongjiang. The different colors represent different combinations of *Hd1*, *Ghd7*, and *DTH7*. N, non-functional haplotypes; W, weak functional haplotypes; F, functional haplotypes.

The accumulated temperature gradually decreased from south to north in Northeast China, while it was divided into three zones in Heilongjiang Province (Supplementary Figure S1B). The different combinations of *Hd1*, *Ghd7*, and *DTH7* were examined in three provinces. The main combination found in Liaoning and Jilin Provinces was NWF, which is generally associated with a long lifespan and high grain yield (**Figure 7A** and **Table 1**). However, NNF plants were mainly distributed in Jilin Province. As expected, the FWF combination did not appear in Heilongjiang Province because of its later heading dates. Thus, we examined the geographical distributions of three-gene combinations in the accumulated temperature zones of Heilongjiang Province. As in Liaoning and Jilin Provinces, the major combination in the first accumulated temperature zone was NWF (**Figure 7A**). The NNN and NWN combinations, associated with a short lifespan and low PS were mainly distributed in the second and third accumulated temperature zones, and particularly in the latter. These results explained why part of Pop 2 had a greater PS index than Pop1 (**Figure 2B**).

To investigate the presence of different gene combinations in rice farming, we examined the gene distributions in cultivars of the top five varieties having planting areas. NWF plants played a core role and accounted for most of the cultivars in Liaoning, Jilin and the first accumulated temperature zone of Heilongjiang (Supplementary Table S13). In addition, NWN and NNN plants were grown as main rice cultivars in the second and third accumulated temperature zones of Heilongjiang, respectively. Notably, ‘Kongyu131,’ which had been planted on over 500,000 hectares a year, was the major rice variety in Heilongjiang Province from 2002 to 2012. After 2013, ‘Longjing31’ was cultivated on over 1 million hectares a year, which is the largest planting area of a *japonica* rice variety in China. This is attributed to the combination NNN of two varieties that are high adaptable, which is affected by the day length and temperature in Heilongjiang Province.

## Discussion

### Japonica Cultivars Are Closely Related in Northeast China

Rice is a staple food and is largely cultivated in Northeast China. In recent decades, rice cultivation areas in Northeast China have increased rapidly because of the good production quality and high economic benefit. It is of both academic and practical value to research the genetic architecture of rice germplasm resources (Xu et al., 2016). Thus, 244 rice materials originating from Northeast China, having a combined 21,198 SNPs that produce high quality genotyping results covering the whole genome, were used to investigate population structures and relationships (Supplementary Figure S1 and Supplementary Table S2). Limited SSR markers have been used in rice classifications in previous studies (Wei et al., 2009; Wang et al., 2014), while a large number of SNPs were used for clustering in this study, increasing the accuracy and reliability of the results. Similar results (low genetic distances and gene diversity levels) were obtained using Powermarker for the population structure analysis, indicating that *japonica* rice cultivars were limited in their hereditary base and had similar genetic backgrounds in Northeast China.

Cultivars have evolved a particular heading behavior in this region after long-term domestication and selection (Li et al., 2015). Since the day length is long and temperature is low, rice cultivars from other regions generally exhibit a long growth period and are unable to adapt to the unique conditions (Wei et al., 2008). As a consequence, the choice of materials is very limited for artificial breeding and it is difficult to introduce the genetic backgrounds of cultivars from other areas into those of the Northeast cultivars. Eventually, this resulted in the close relationship among *japonica* rice cultivars in Northeast China. The cultivars of Pop 2, which mostly originated from Liaoning Province, exhibited slight higher levels of variability (**Figure 1**). The new genes from the rich biological resources of the southern region were easily introduced to the cultivars of Liaoning, which is geographically closer to the southern region.

For the GWAS, the population structure, which widely existed in the natural groups, usually increased linkage disequilibrium values between chromosomes, leading to false positives (Rafalski and Morgante, 2004). The relationships between individuals also had an impact on the results of the association analysis (Flint-Garcia et al., 2003, 2005). A close relationship of the population in this study exhibiting large extent of linkage disequilibrium may cause numerous SNPs associations on the different chromosomes. It is difficult to identify unknown genes owing to many candidate genes in a linkage disequilibrium block. But the QTLs with known genes can be easily identified in a local population with low genetic complexity and the genetic architecture (Fujino et al., 2015). The SNP associations may generate spurious associations between the phenotype and unlinked SNPs. According to a previous report, an association model with Q and K as covariates was more able than other models to reduce the rates of false positives (Yu et al., 2006). Likewise, the MLM was significantly better than the GLM in controlling corrections in this study (Supplementary Figure S5). Although false positives were well controlled, the MLM produced false negatives. A close relationship with strict parameters may cause true loci to be eliminated in the MLM. Thus, the MLM was regarded as an ideal model to reduce false positive associations, while the GLM was necessary for the GWAS to avoid false negatives.

### *Hd1*, *Ghd7*, and *DTH7* Combinations Determined the Different Rice Growing Regions

Grain yield is positively correlated with heading date, which is a vital factor in controlling rice yield (Gao et al., 2014). Varieties with an appropriate heading date can fully utilize the light and temperature resources to improve grain yields. *Ghd7* and *DTH7* play important roles in rice adaptation to LD conditions by reducing PS and causing early flowering (Xue et al., 2008; Koo et al., 2013). *Hd1*, a major PS quantitative trait locus, functions as a floral suppressor by forming a complex with *Ghd7* and *DTH7*, although *Ghd7* and *DTH7* can delay flowering without the functional *Hd1* allele under LD conditions (Yano et al., 2000; Nemoto et al., 2016; Goretti et al., 2017). In this study, *Ghd7* and *DTH7* as major repressors of flowering were detected by the GWAS (**Figures 5**, **6**). Asian cultivated rice, which contains functional alleles of *Hd1*, *Ghd7*, and *DTH7* that have strong effects, originated from low-latitude regions with SD lengths. After long-term selection and domestication, the alleles of *Hd1*, *Ghd7*, and *DTH7* with weak or no functions were generated to reduce the effects of prolonging heading in high-latitude regions with a LD lengths (**Table 1** and Supplementary Table S12).

When rice was expanded to the most northerly regions, varieties harboring NNN were selected owing to their early heading dates and lack of PS (**Figure 7**). ‘Kongyu131’ and ‘Longjing31,’ carrying NNN, are mainly grown in the third accumulated temperature zone and possess the largest cropping area per year in China (Supplementary Table S13). FNN, NWN, and FWN plants showing phenotypes similar to that of NNN plants were widely grown in the second and third accumulated temperature zones. This phenomenon was because *Hd1* had no function by itself and *Ghd7* had a weak function. The varieties having NWF were widely planted in Northeast China because they had suitable flowering times that allow them to fully utilize the light and temperature resources. FWF plants, which exhibited a long flowering time, were mainly grown south of Liaoning. To summarize, loss-of-function alleles of *Hd1*, *Ghd7*, and *DTH7* existed in the rice grown in northerly regions because of their association with early heading dates, while functional alleles of *Hd1*, *Ghd7*, and *DTH7* existed in rice grown in southerly regions because of their association with late heading dates in Northeast China.

### Selection of Flowering Genes as a Tool for Breeding

Heading date is a complex trait and is seriously affected by environmental factors (Sun et al., 2014). Local varieties are usually adapted to local environments, including proper heading dates. The functions of *Hd1*, *Ghd7*, and *DTH7* were clear, and their effects could be additive. Therefore, predicting heading dates in hybrid progenies could be possible based on their parental varieties. For instance, no significant segregation should be observed in the cross progenies of two parents with the same combination of these three genes. The late heading of F_1_ hybrids might occur, and progeny will be different from the two parents with complementary *Hd1*, *Ghd7*, and *DTH7* genes. For example, an FWN plant crossed with an NNF plant. The optimized heading date could be designed using combinations of these three genes. The optimum combination would be selected to match the main cultivars in the locality. For instance, NNN plants would be selected in the third accumulated temperature zone.

The genetic diversity of varieties can improve the resistance of cultivars to disease (Zhu et al., 2000). Generally, the goals of crop breeding are high yield and quality to meet the supply demands, but this reduces the genetic diversity of the population, which, in turn, decreases the ability of crops to respond to stress. The long-term major aim of breeders in Northeast China is to produce higher and higher yields, resulting in many problems, including the loss of genetic diversity. In addition, the unique climate and single variety type (single-season rice) may lead to reductions in genetic diversity. Introducing genes from varieties in other regions into the Northeast *japonica* varieties may result in a sustained development of rice production. The early *indica* rice, which was mainly cultivated in the Yangtze region and southern China, exhibits no or low PS and has a short growth duration. Part of cultivars exhibited no PS genes, having mutations in *Hd1*, *Ghd7*, and *DTH7*, while the remaining cultivars carried either functional *Hd1* or *Ghd7* genes (Xu et al., 2010). Thus, in terms of heading date, it is a reasonable strategy to breed new cultivars by selecting progenies from Northeast *japonica* rice and early *indica* rice, which can broaden the genetic base of the breeding materials and improve the genetic diversity of rice varieties in Northeast China.

Obvious heterosis has been found among *indica* × *japonica* crosses, which represent an important strategy to enhance rice yield potentials (Zheng and Yong Gen, 1999). The late heading dates in *indica* × *japonica* F_1_ hybrids occur because of interactions between the heading date-related genes of the two parents, such as *Hd1* and *Ghd7* (Wei et al., 2010a). Heterosis utilization may become a reality in Northeast China, with one major problem: the long heading dates of F_1_ hybrids. Thus, two parents with the same heading date-related gene or no PS gene (e.g., combination NNN) may be the key to obtaining suitable heading stages in F_1_ hybrids of *indica* × *japonica*. Introducing early *indica* rice, and particularly those cultivars with no PS, might facilitate heterosis utilization in Northeast China. Those could provide guidance for molecular designed breeding of rice with specific heading date.

## Author Contributions

JY, XN, and XW: designed the research experiments. JY, YF, and ShaW: performed the phenotyping. QX, YF, and ShuW: carried out the genetic studies. XY, HY, and YW: managed the materials. XW: designed the overall project. JY and XN: analyzed the data and drafted the manuscript. ShuW and YF: helped to revise the manuscript. All of the authors read and approved the final manuscript.

## Conflict of Interest Statement

The authors declare that the research was conducted in the absence of any commercial or financial relationships that could be construed as a potential conflict of interest.

## Footnotes

1 http://rice.plantbiology.msu.edu/pub/data/Eukaryotic_Projects/o_sativa/annotation_dbs/pseudomolecules/version_6.0/

2 http://rice.plantbiology.msu.edu/index.shtml

3 http://rapdb.dna.affrc.go.jp/

4 http://www.toyobo-global.com

5 http://www.takara.com.cn/

6 http://www.appliedbiosystems.com/
