# Supplementary material for: Divergent Hd1, Ghd7, and DTH7 Alleles Control Heading Date and Yield Potential of Japonica Rice in Northeast China
Source: Front Plant Sci. 2018 Jan 26;9:35. doi: 10.3389/fpls.2018.00035 (PMC5790996; doi:10.3389/fpls.2018.00035)
Supplement: Supplementary file 3 [file Table_3.DOCX]

**TABLE S3 | Primers used in this study.**

| Primers | Forward 5’- 3’ | Reverse 5’- 3’ | Reference |
| --- | --- | --- | --- |
| *Hd1*-1 | gtgactttcccctccctagc | agcctaaagatcgcagcttg |  |
| *Hd1*-2 | acagcaatcaccacacgaaa | tccggaaattacaaagcaaaa |  |
| *Ghd7*-1 | ctttccctcatccccaactt | agacgaggaggatcgatgaa |  |
| *Ghd7*-2 | tgcatttgcttatgcgtaca | aatgggccatcgatcactaa |  |
| *DTH7* | tgtcgctagtgagttaggaaacc | tcgatcggccaaggaagaagaat |  |

**TABLE S5 |Population differentiation statistics between pairwise subpopulations.**

|  | Pop1 | Pop2 | Mixed |
| --- | --- | --- | --- |
| Pop1 | 0 | 0.100 | 0.032 |
| Pop2 | 0.100 | 0 | 0.046 |
| Mixed | 0.032 | 0.046 | 0 |

**TABLE S6 | Genetic diversity analysis of different subpopulations.**

| Population | Major Allele Frquency | Gene Diversity | Heterozygosity | Polymorphic information content |
| --- | --- | --- | --- | --- |
| Pop1 | 0.823 | 0.249 | 0.004 | 0.207 |
| Pop2 | 0.797 | 0.286 | 0.002 | 0.234 |
| Mixed | 0.802 | 0.284 | 0.001 | 0.235 |
| Totality | 0.797 | 0.292 | 0.003 | 0.243 |

**TABLE S7 | Pearson’s correlation coefficient for heading dates in four environments.**

|  | 2015Lingshui | 2016Lingshui | Hangzhou | Shenyang |
| --- | --- | --- | --- | --- |
| 2015Lingshui | 1 |  |  |  |
| 2016Lingshui | 0.803 | 1 |  |  |
| Hangzhou | 0.572 | 0.689 | 1 |  |
| Shenyang | 0.494 | 0.612 | 0.805 | 1 |

**TABLE S8 |Two-way analysis of variance for heading dates in four environments.**

| Source of variance | df | SS | SSG/SST (%) |
| --- | --- | --- | --- |
| G | 243 | 154799.55 | 15.89 |
| E | 3 | 761653.89 | 78.15 |
| G×E | 729 | 58114.10 | 5.96 |

G genotype, e environment; G×E, Interaction of genotype and environment; SSG, genotype sum-of-squares; SST, total sum-of-squares.

**TABLE S9 | Correlation and path analyses between yield characteristics and heading dates in Shenyang.**

| Factor | Correlation coefficient with yield | Direct path coefficient | Indirect path coefficient | | | |
| --- | --- | --- | --- | --- | --- | --- |
|  |  |  | Panicles per plant | Spikelets per panicle | Thousand kernel weight | Days to heading |
| Panicles per plant | 0.3026 | 0.5150 |  | -0.1732 | -0.0199 | -0.0193 |
| Spikelets per panicle | 0.7225 | 0.6887 | -0.1295 |  | 0.0076 | 0.1557 |
| Thousand kernel weight | 0.1797 | 0.1710 | -0.0601 | 0.0307 |  | 0.0381 |
| Days to heading | 0.6842 | 0.2272 | -0.0436 | 0.4720 | 0.0286 |  |

**TABLE S10 | Physical positions of nine SNPs** marginally **significantly associated with heading date under the three environmental conditions.**

| Condition | Marker | Chromosome | Physical position | *P* | R^2^ | Known locus |
| --- | --- | --- | --- | --- | --- | --- |
| Shenyang | F0101175090 | 1 | 1175090 | 1.12E-05 | 0.08312 |  |
| Shenyang | R0610460215 | 6 | 10460215 | 3.61E-05 | 0.07318 | *Hd1* |
| Hangzhou | R0620151786 | 6 | 20151786 | 1.20E-05 | 0.09855 |  |
| Hangzhou | F0711913681 | 7 | 11913681 | 1.57E-05 | 0.08465 | *Ghd7* |
| Hangzhou | F0804960931 | 8 | 4960931 | 3.16E-06 | 0.09509 |  |
| Hangzhou | F0909163222 | 9 | 9163222 | 5.83E-06 | 0.10447 |  |
| Hangzhou | F1127320094 | 11 | 27320094 | 1.91E-05 | 0.07803 |  |
| Hangzhou | R1200352430 | 12 | 352430 | 1.54E-06 | 0.09962 |  |
| Lingshui | R0611892216 | 6 | 11892216 | 2.12E-05 | 0.07757 | *Hd1* |

| Genes (bp) | Provinces | S | π (10^-3^) | θ(10^-3^) | TD | FD | haplotype  diversity |
| --- | --- | --- | --- | --- | --- | --- | --- |
| *Hd1*(1224) | All | 10 | 4.41 | 1.49 | 4.48045^***^ | 1.31584 | 0.527 |
|  | Heilongjiang | 14 | 5.52 | 2.36 | 3.72786^***^ | 1.55021^*^ | 0.464 |
|  | Jilin | 14 | 4.92 | 3.11 | 1.99579 | 1.51358^*^ | 0.465 |
|  | Liaoning | 10 | 3.84 | 1.67 | 3.21556^**^ | 1.36068 | 0.492 |
| *Ghd7*(774) | All | 4 | 0.36 | 0.85 | -1.00635 | -0.44509 | 0.249 |
|  | Heilongjiang | 1 | 0.55 | 0.26 | 1.34001 | 0.50129 | 0.424 |
|  | Jilin | 3 | 0.62 | 1.02 | -0.96529 | -1.40049 | 0.385 |
|  | Liaoning | 4 | 0.08 | 0.96 | -1.74635 | -4.01282 | 0.032 |
| *DTH7*(2229) | All | 5 | 0.22 | 0.37 | -0.78306 | 0.96484 | 0.417 |
|  | Heilongjiang | 5 | 0.25 | 0.45 | -0.94139 | -2.08422 | 0.471 |
|  | Jilin | 2 | 0.07 | 0.24 | -1.51314 | -2.20378 | 0.151 |
|  | Liaoning | 4 | 0.06 | 0.33 | -1.58267 | 0.92249 | 0.063 |

**TABLE S11 | Nucleotide diversity analysis and the neutrality test.**

S, segregation sites; π, average number of nucleotide differences per site between random

two sequences; θ, Watterson estimator; TD, Tajima's D, FD, Fu and Li’s test D. *, P < 0.05; **, P < 0.01; ***, P < 0.001.

| Gene | Hap. | Reported | Mutation | Fun | Reference |
| --- | --- | --- | --- | --- | --- |
| *Hd1* | Hap.A | Type 6; Hap.A |  | F | Takahashi etal. (2009); Yano etal.(2016) |
|  | Hap.B | Hap.B |  | F | Yano etal.(2016) |
|  | Hap.C |  | -2 bp | N |  |
|  | Hap.D |  | -43 and -2 bp | N |  |
|  | Hap.E |  | -43 bp | N |  |
|  | Hap.F | Hap.E | -43 bp | N | Yano etal.(2016) |
|  | Hap.G | Hap.F | -43 bp | N | Yano etal.(2016) |
|  | Hap.H | Hap.D |  | F | Yano etal.(2016) |
|  | Hap.I | Type 9 | -33 bp | F | Takahashi etal. (2009) |
|  | Hap.J | Type 10 | -33 and +150 bp | F | Takahashi etal. (2009) |
|  | Hap.K |  |  | F |  |
|  | Hap.L | Type 14 | -2 bp | N | Takahashi etal. (2009) |
|  | Hap.M | Type 11 | -33 and +156 bp | F | Takahashi etal. (2009) |
|  | Hap.N | hap11 | -33 and +312bp | N | Zhang etal. (2008) |
| *Ghd7* | Hap.A |  |  | F |  |
|  | Hap.B | Ghd7-1 |  | S | Xue etal. (2008) |
|  | Hap.C | Ghd7-2 |  | W | Xue etal. (2008) |
|  | Hap.D | Ghd7-0a | Premature stop | N | Xue etal. (2008) |
| *DTH7* | Hap.A |  |  | U |  |
|  | Hap.B | *PRR37-2* |  | F | Koo etal.(2013) |
|  | Hap.C | *PRR37-2a* |  | N | Koo etal.(2013) |
|  | Hap.D | *PRR37-1* |  | F | Koo etal.(2013) |
|  | Hap.E | *PRR37-1a* | -8 bp | N | Koo etal.(2013) |

**TABLE S12 | Functional classifications of *Hd1*, *Ghd7* and *DTH7* alleles in rice.**

F, N, S and W represent functional, nonfunctional, strong and weak alleles, respectively. Negative and positive fragment lengths in column mutation signify fragment deletion and fragment insertion.

**TABLE S13 | Top five planting area of varieties in Northeast China.**

| Variety | Combinations | | | Province | Accumulated  temperature zone | Planting  area in 2013 |
| --- | --- | --- | --- | --- | --- | --- |
|  | *Hd1* | *Ghd7* | *DTH7* |  |  |  |
| Songjing6hao | N | N | F | Heilongjiang | Ⅰ | 974 |
| Songjing9hao | N | W | F | Heilongjiang | Ⅰ | 866 |
| Wuyoudao1hao | N | W | F | Heilongjiang | Ⅰ | 847 |
| Kendao10hao | N | W | F | Heilongjiang | Ⅰ | 542 |
| Songjing12 | N | W | F | Heilongjiang | Ⅰ | 463 |
| Kendao12hao | N | W | N | Heilongjiang | Ⅱ | 1816 |
| Hejiang23hao | N | W | N | Heilongjiang | Ⅱ | 1189 |
| Longjing21hao | N | N | N | Heilongjiang | Ⅱ | 1163 |
| Suijing9hao | N | W | N | Heilongjiang | Ⅱ | 659 |
| Longjing12hao | N | W | N | Heilongjiang | Ⅱ | 577 |
| Longjing31hao | N | N | N | Heilongjiang | Ⅲ | 2458 |
| Longjing20hao | F | N | N | Heilongjiang | Ⅲ | 869 |
| Kongyu131 | N | N | N | Heilongjiang | Ⅲ | 578 |
| Longjing27hao | F | N | N | Heilongjiang | Ⅲ | 443 |
| Longjing13hao | N | N | N | Heilongjiang | Ⅲ | 282 |
| Jijing88 | N | W | F | Jilin |  | 531 |
| Jiudao58hao | N | W | F | Jilin |  | 109 |
| Jijing62hao | F | W | F | Jilin |  | 101 |
| Jinongda808 | N | W | F | Jilin |  | 41 |
| Jinongda23hao | N | W | F | Jilin |  | 21 |
| Yanfeng47 | N | W | F | Liaoning |  | 1846 |
| Liaojing294 | N | W | F | Liaoning |  | 1670 |
| Liaojing454 | N | W | F | Liaoning |  | 1276 |
| Liaoxing1hao | N | W | F | Liaoning |  | 906 |
| Liaojing9hao | N | W | F | Liaoning |  | 801 |
