# Supplementary material for: Divergent Hd1, Ghd7, and DTH7 Alleles Control Heading Date and Yield Potential of Japonica Rice in Northeast China
Source: Front Plant Sci. 2018 Jan 26;9:35. doi: 10.3389/fpls.2018.00035 (PMC5790996; doi:10.3389/fpls.2018.00035)
Supplement: Supplementary file 5 [file Image_1.pdf]

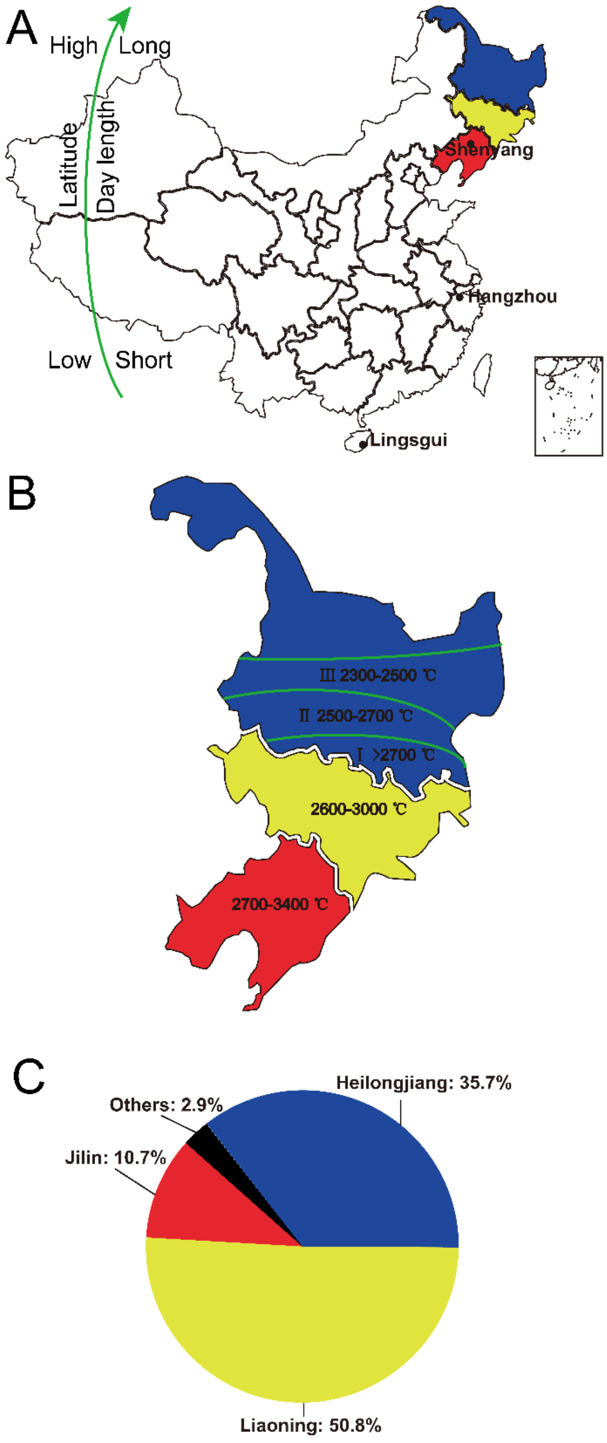

Fig. S1. Diagram of rice cultivation in Northeast China. (A) Map of China. Geographical locations of three planting stations, Lingshui (18°32'N, 110°01'E), Hangzhou (30°15' N, 120°12' E) and Shenyang (41°48'N, 123°25'E). The positions of the Heilongjiang, Jilin and Liaoning are highlighted with blue, yellow and red, respectively. (B) The accumulated temperatures in Northeast China. The accumulated temperatures decrease from south to north, and rice cultivation was divided into three accumulated temperature zones in Heilongjiang. (C) Proportion of the 244 varieties grown in the different zones.

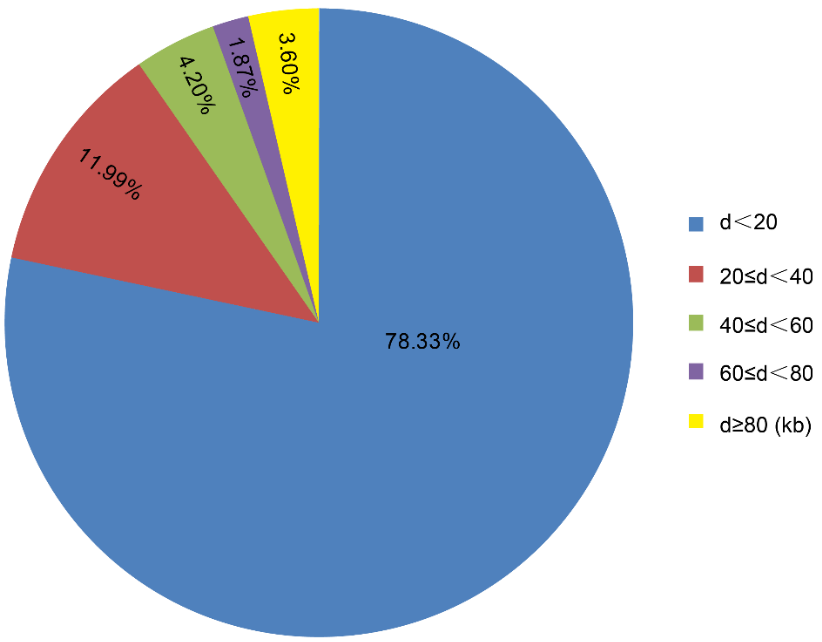

Fig. S2. Proportion of the 21,198 SNPs categorized by inter-SNP (the adjacent SNPs) distances. d represents the distance between two adjacent SNPs.

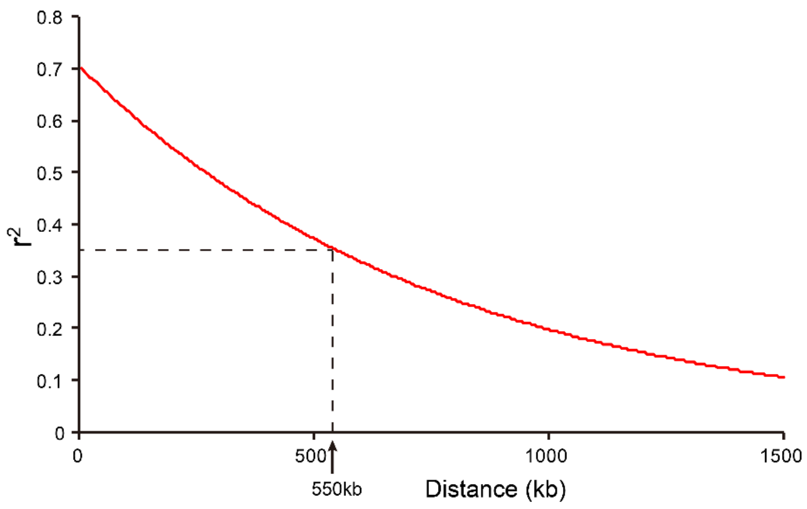

Fig. S3. Estimation of genome-wide average linkage disequilibrium decay distances.

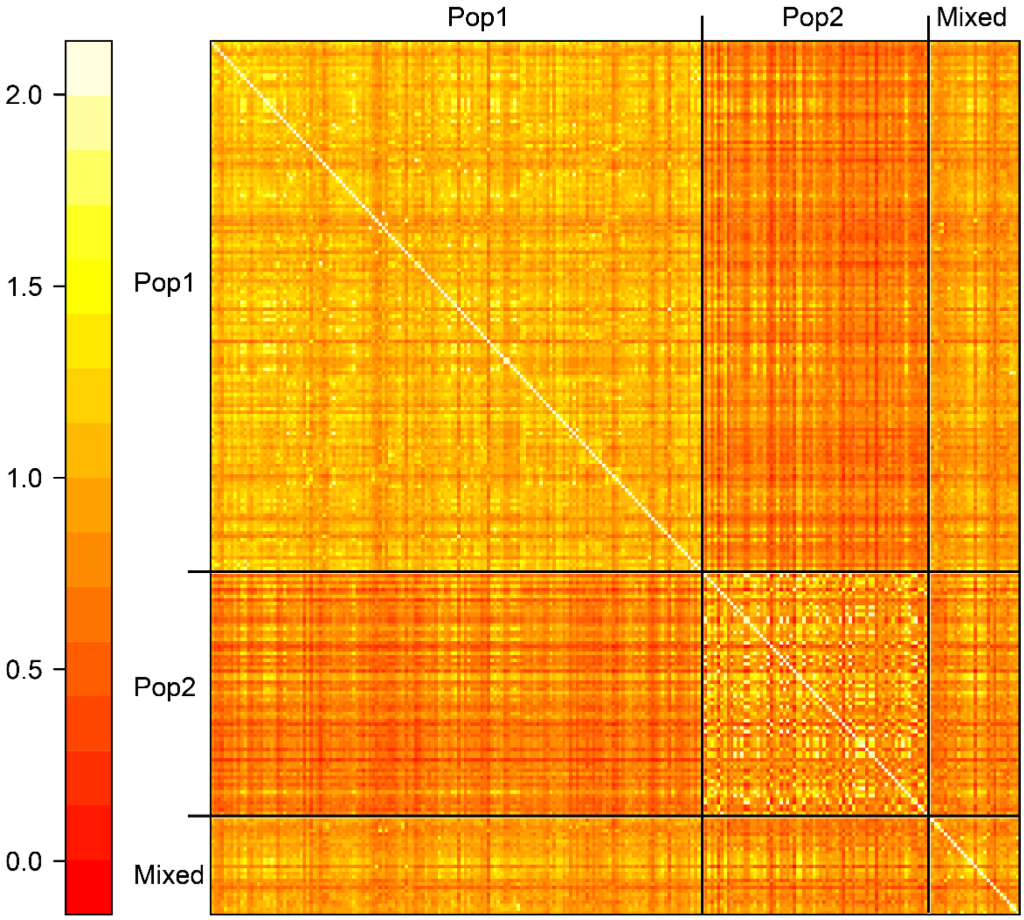

Fig. S4. Heatmap of pairwise relative kinship values. Each pixel in the square indicates the range of kinship values as shown in the left-hand colored legend.

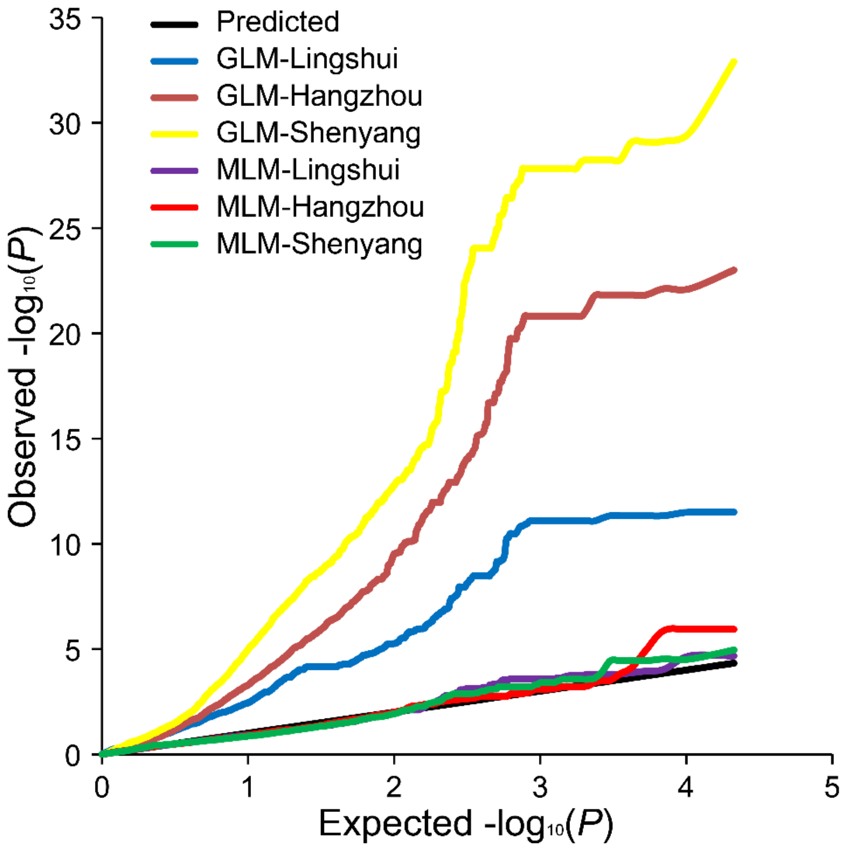

Fig. S5. Quantile–quantile plots for heading dates in two models.

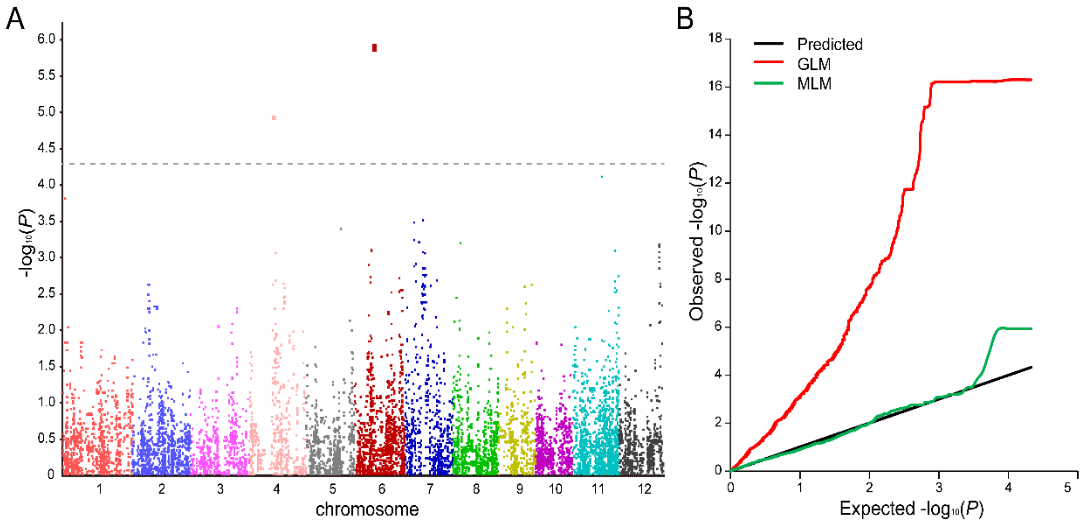

Fig. S6. GWAS heading date in Lingshui in 2016. (A) GWAS results using a mixed linear model. (B) Quantile–quantile plots for heading dates in two models.

A

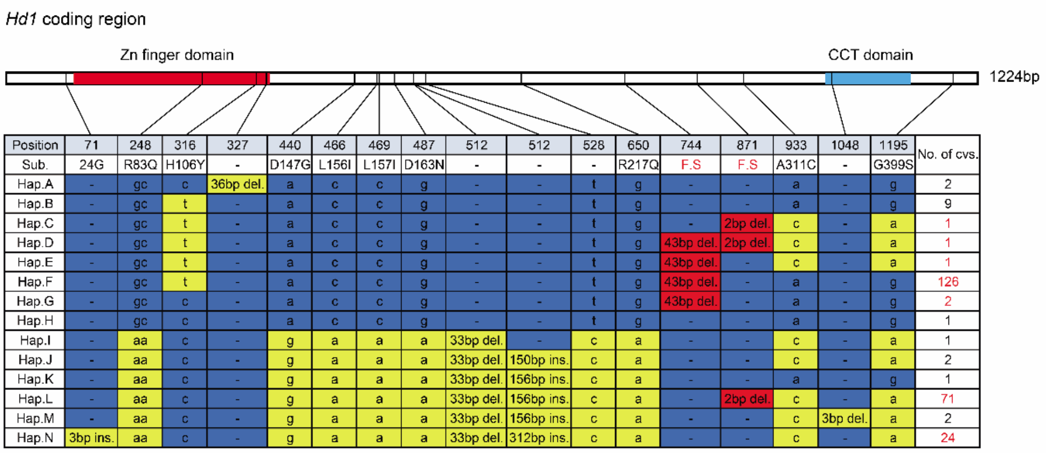

Position	71	248	316	327	440	466	469	487	512	512	528	650	744	871	933	1048	1195	No. of cvs.
Sub.	24G	R83Q	H106Y	-	D147G	L156I	L157I	D163N	-	-	-	R217Q	F.S	F.S	A311C	-	G399S	
Hap.A	-	gc	c	36bp del.	a	c	c	g	-	-	t	g	-	-	a	-	g	2
Hap.B	-	gc	t	-	a	c	c	g	-	-	t	g	-	-	a	-	g	9
Hap.C	-	gc	t	-	a	c	c	g	-	-	t	g	-	2bp del.	c	-	a	1
Hap.D	-	gc	t	-	a	c	c	g	-	-	t	g	43bp del.	2bp del.	c	-	a	1
Hap.E	-	gc	t	-	a	c	c	g	-	-	t	g	43bp del.	-	c	-	a	1
Hap.F	-	gc	t	-	a	c	c	g	-	-	t	g	43bp del.	-	a	-	g	126
Hap.G	-	gc	c	-	a	c	c	g	-	-	t	g	43bp del.	-	a	-	g	2
Hap.H	-	gc	c	-	a	c	c	g	-	-	t	g	-	-	a	-	g	1
Hap.I	-	aa	c	-	g	a	a	a	33bp del.	-	c	a	-	-	c	-	a	1
Hap.J	-	aa	c	-	g	a	a	a	33bp del.	150bp ins.	c	a	-	-	c	-	a	2
Hap.K	-	aa	c	-	g	a	a	a	33bp del.	156bp ins.	c	a	-	-	a	-	g	1
Hap.L	-	aa	c	-	g	a	a	a	33bp del.	156bp ins.	c	a	-	2bp del.	c	-	a	71
Hap.M	-	aa	c	-	g	a	a	a	33bp del.	156bp ins.	c	a	-	-	c	3bp del.	a	2
Hap.N	3bp ins.	aa	c	-	g	a	a	a	33bp del.	312bp ins.	c	a	-	-	c	-	a	24

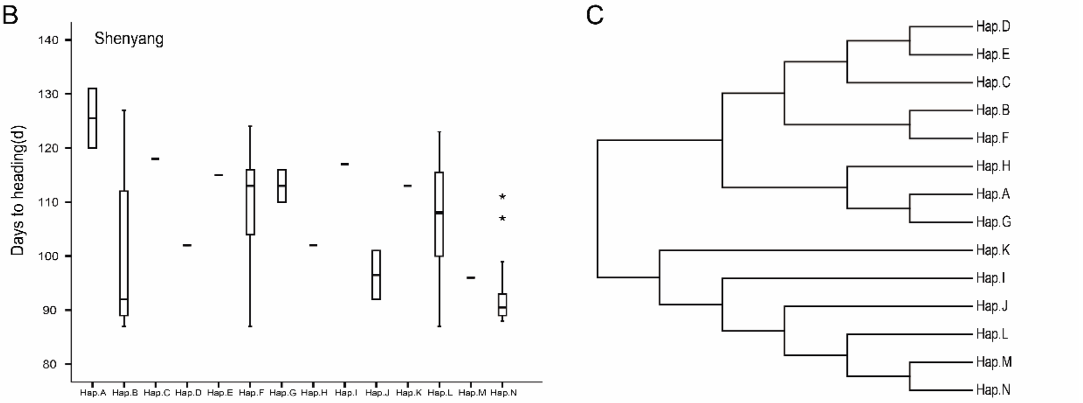

Fig. S7. A high degree of polymorphism in the *Hd1* coding sequence, and its haplotypes with flowering times. (A) Haplotype analysis of *Hd1*. The sites, which cause frame-shift mutations or create premature stop codons, are marked in red. (B) Boxplots for days to heading based on the haplotypes of *Hd1*. (C) Unrooted phylogenetic tree of *Hd1*.

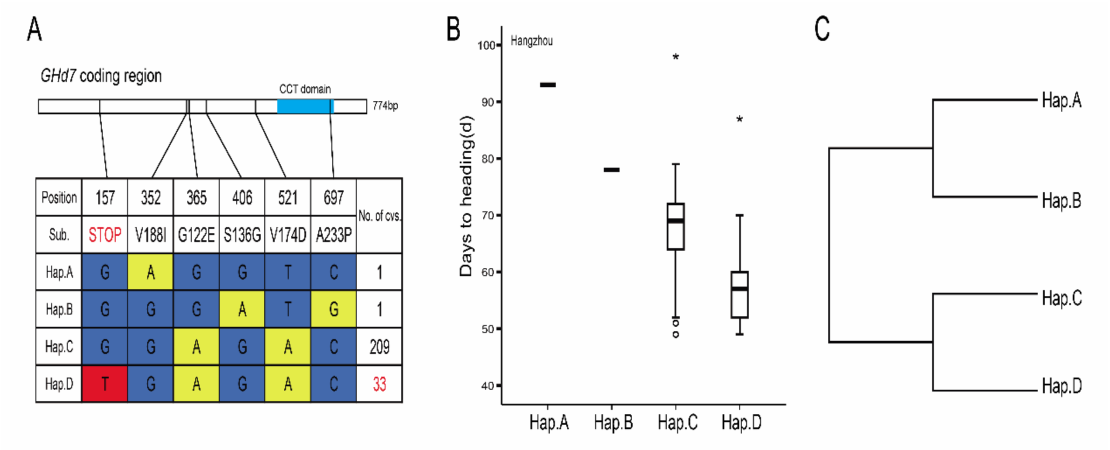

Fig. S8. High degree of polymorphism in the *Ghd7* coding sequence, and its haplotypes with flowering times. (A) Haplotype analysis of *Ghd7*. (B) Boxplots for days to heading based on the haplotypes of *Ghd7*. (C) Unrooted phylogenetic tree of *Ghd7*.

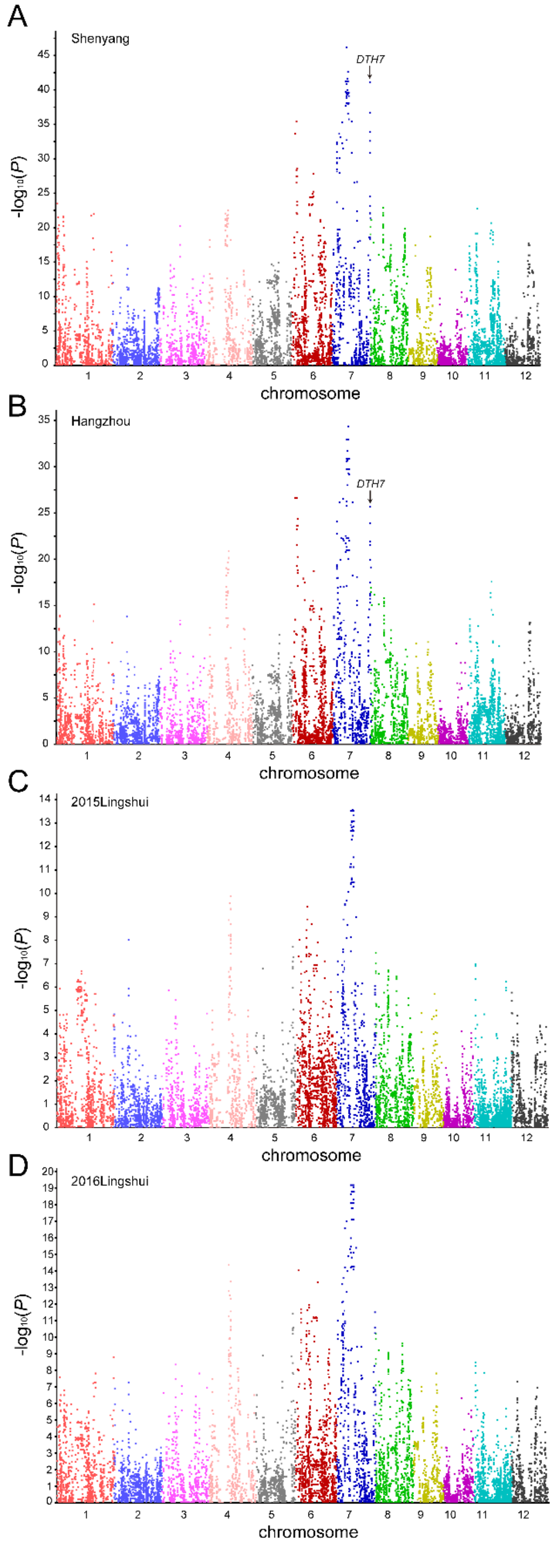

Fig. S9. Manhattan plots of general linear model under four conditions. (A) Shenyang. (B) Hangzhou. (C) 2015Lingshui. (D) 2016Lingshui.

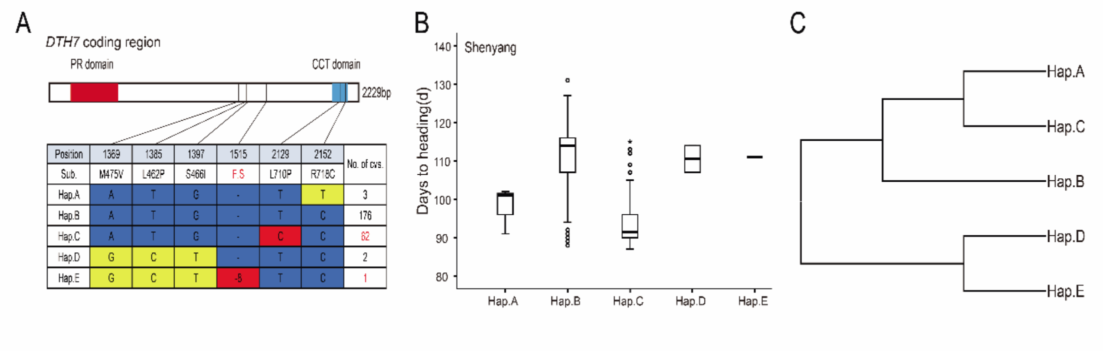

Fig. S10. A high degree of polymorphism in the *DTH7* coding sequence, and its haplotypes with flowering times. (A) Haplotype analysis of *DTH7*. (B) Boxplots for days to heading based on the haplotypes of *DTH7*. (C) Unrooted phylogenetic tree of *DTH7*.
